# Monitoring the emergence of antibiotic resistance using the technology ot the DebugIT platform in the HEGP context

**DOI:** 10.1186/1753-6561-5-S6-P320

**Published:** 2011-06-29

**Authors:** R Choquet, C Daniel, P Grohs, N Douali, M-C Jaulent

**Affiliations:** 1 UMR_S 872, EQ20, INSERM, France; 2 Service de microbiologie, HEGP -APHP, PARIS, France

## Introduction / objectives

This work takes part in the European DebugIT project which goal is to build a technical and semantic information technology platform able to share heterogeneous clinical data sets from different hospitals for the monitoring and the control of infectious diseases and antimicrobial resistances in Europe. The aim of the study is to compare the incidence rates of antimicrobial resistance at the HEGP hospital obtained in real-time by the DebugIT platform to those established by the yearly-performed analysis processed by the microbiologists of the HEGP hospital.

## Methods

The INSERM database covers seven years of anonymized microbiology data and represents an image of the HEGP EHR data. To be able to semantically integrate the data with other European peers, we went through several steps of data normalisation and quality works. These tasks led to the setup of semantic data providers (hospitals) that were integrated at a European level. We built a common view of our domain knowledge upon which we aligned our semantic data providers. We compared the incidence rates of antimicrobial resistance produced by the DebugIT platform at the HEGP hospital to a gold standard produced yearly by the experts.

## Results

Despite different data processing methods (e.g only microbiologists de-duplicate data in case of repetitive antibiograms on different isolates), the results were highly similar (maximum 2% variability of the antimicrobial resistance incidence rates).

## Conclusion

This study shows the adequacy of the control capabilities of the DebugIT platform and the maturity of the semantic integration methods developed by the project consortium for the setup of a pan-European surveillance network.

## Disclosure of interest

None declared.

